# Tuberculose pulmonaire révélée par un purpura thrombopénique chez l'enfant-à propos d'un cas clinique observé au service de pédiatrie des Cliniques Universitaires de Lubumbashi

**Published:** 2012-07-17

**Authors:** Toni Kasole Lubala, Augustin Mulangu Mutombo, Arthur Ndundula Munkana, Michel Muteya Manika

**Affiliations:** 1 Cliniques Universitaires De Lubumbashi, Département De Pédiatrie BP 1825, Lubumbashi, RDC; 2 Cliniques Universitaires De Lubumbashi, Service D'Imagerie Médicale BP 1825, Lubumbashi, RDC; 3 Cliniques Universitaires De Lubumbashi, Service Immuno-Hematologie Et Transfusion BP 1825, Lubumbashi, RDC

**Keywords:** Purpura thrombopénique, tuberculose, corticothérapie, enfant, thrombocytopenic purpura, tuberculosis, corticotherapy, child

## Abstract

Nous rapportons le cas d'un enfant de 7 ans, de sexe masculin ayant présenté un purpura thrombopénique avec épistaxis, hématémèse, otorragies et pétéchies généralisées. Durant la même hospitalisation, nous avons mis en évidence une tuberculose pulmonaire documentée par la présence de bacilles acido-alcoolo résistants à l'examen des crachats. Nous avons observé une majoration du taux de plaquettes en une semaine de corticothérapie intraveineuse à haute dose, avant l'instauration d'une poly chimiothérapie antituberculeuse. Nous rappelons également la controverse que suscite la prise en charge de cette association rarement rapportée.

## Introduction

Des troubles hématologiques sont couramment observés dans la tuberculose active [1]. Cependant, les thrombopénies sont rares et les purpuras thrombopéniques associés à la tuberculose ne sont mentionnés que dans très peu de publications. Pourtant, il y a dans le monde d'après l'OMS, 8 millions de cas annuels de tuberculose et deux millions de décès dus à cette maladie. On estime qu'un tiers de l'humanité est infecté par le bacille de Koch (BK). Les cas de tuberculose augmentent de 1 à 2% par an. Nous rapportons le cas d'une tuberculose disséminée révélée par un syndrome hémorragique chez un enfant de 7 ans.

## Patient et observation

Un enfant de 7 ans de sexe masculin a été reçu aux urgences en décembre 2009 pour épistaxis, otorragie, hématémèse et méléna survenus ce jour dans un contexte a fébrile. Les parents rapportent également une discrète toux productive depuis plusieurs mois pour laquelle ils ont consulté plusieurs fois sans qu'aucune amélioration ne soit observée.

**Figure 1:**
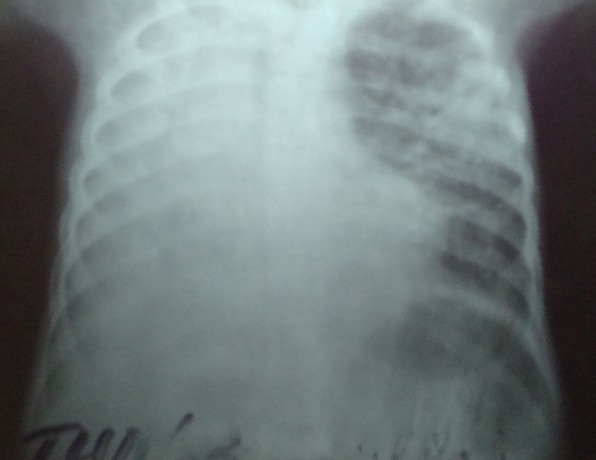
Radiographie du thorax incidence face prise en décubitus dorsal à J1 d'hospitalisation montrant une pleuropneumonie droite ainsi qu'un foyer lobaire supérieur gauche

Le patient est né à terme, d'une grossesse sans particularité. Il n'a pas d'antécédent médical particulier. Vacciné par le BCG en période néonatale selon le calendrier vaccinal en vigueur en république démocratique du Congo. L'anamnèse met en évidence une notion de contage il y a deux ans avec ses grands-parents ainsi que chez un oncle paternel dont les crachats ont mis en évidence des bacilles acido-alcoolo-résistants. Le patient pèse 14,4 kg (<P3) et mesure 104 cm (P3-P10). Sa courbe de croissance staturo-pondérale n'a pas pu être établie faute de mesures antérieures fiables. A son admission, il est tachycarde (160 bpm), tachypnéique (48 cpm) et présente une température axillaire de 36 degrés Celsius. Son état général était altéré, ses conjonctives palpébrales pales et bulbaires anictériques. On objective une otorragie bilatérale, une épistaxis ainsi que des plaques pétéchiales sur le palais ainsi que sur la face antérieure du thorax. Deux ganglions supra centimétriques, mobiles, non sensibles sont palpés en région sous-maxillaire. L'examen oto-rhino-laryngologique met en évidence une hypoacousie ainsi qu'une perforation tympanique bilatérale ainsi qu'un saignement à travers les membranes tympaniques perforées. On observe un discret tirage intercostal ainsi qu'une déformation du thorax d'apparition progressive selon les parents, avec une attitude scoliotique modérée. L'auscultation est asymétrique, avec une nette diminution de l'entrée d'air dans tout le poumon droit. Des crépitant lointains sont perçus au poumon droit. L'auscultation du cœur est sans particularité. Il présente une hépatomégalie à 2 cm, non sensible, à surface lisse, sans reflux hépato-jugulaire associé. L'examen des membres supérieurs et inférieurs est marque par un “clubbing”. Une cicatrice laissée par le BCG est présente à la face antérieure de l'avant-bras gauche. Le bilan para clinique initial met en évidence une thrombopénie isolée a 90000/mm3 et un temps de saignement allongé (5 min 30 sec). Le reste des examens utiles au décryptage des troubles de l'hémostase n'ont pas été réalisés parce que non disponibles à Lubumbashi. Le bilan inflammatoire montre une accélération de la vitesse de sédimentation à 110 mm à la première heure, une numération des globules blancs à 7200/mm3 avec une formule leucocytaire mixte. Le test sérologique rapide à la recherche d'anticorps anti-VIH était négatif. La radiographie du thorax met en évidence une pleuropneumonie droite importante ainsi qu'un foyer lobaire supérieur gauche (Figure 1). Le diagnostic de purpura thrombopénique dans un contexte de broncho-pneumopathie d'allure chronique est posé et l'hypothèse d'une tuberculose évoquée. L'anémie est corrigée en urgence par une transfusion de sang total frais, une corticothérapie par dexamethazone 3 mg/kg /j et une antibiothérapie par ceftriaxone iv démarrée dans l’éventualité d'un purpura infectieux. On observe une majoration du taux de plaquettes (120000/mm3) ainsi qu′un amendement du syndrome hémorragique à J7 d'hospitalisation. Le bilan para clinique complété par l'examen des crachats par la technique de zhiel-Nielsen met en évidence a J7 d'hospitalisation, des bacilles acido-alcoolo-résistants dans trois prélèvements consécutifs. L'intradermoréaction à la tuberculine n'a pas été réalisée faute d'intrants. L’échographie abdominale ne met en évidence ni adénopathie dans les territoires des gros vaisseaux, ni ascite. Le diagnostic de tuberculose pulmonaire est posé et une poly chimiothérapie antituberculeuse démarrée en remplacement de la ceftriaxone.

## Discussion

Le purpura thrombopénique associé à la tuberculose est une entité extrêmement rare tant chez l'enfant que chez l'adulte. Une étude réalisée il y a dix ans en Arabie Saoudite a révélé que le purpura thrombopénique était observé dans 1 pourcent des cas de tuberculose active retenus dans leur échantillon [1].

L'un des premiers cas clinique de purpura thrombopénique chez l'enfant associe a la tuberculose pulmonaire rapporté dans la littérature a été observé en Turquie et rapporté en 2009. Il s'agissait d'un purpura thrombopénique immunitaire révélé par des épistaxis importantes ainsi que par des pétéchies généralisées [2]. Dans notre observation, une majoration de la numération des plaquettes a été obtenue avant le début de la poly chimiothérapie antituberculeuse. Celle-ci a été déterminante dans la restauration du taux de plaquettes dans le cas observé en Turquie [2].

Une surdité liée à un hemotympan bilatéral sans perforation des membranes tympanique a été rapporte chez un enfant de 5 ans en Turquie dans le contexte d'un purpura thrombopénique immun [3]. Chez notre patient, une hypoacousie liée à un hemotympan bilatéral perforé spontanément a été observé.

Nous n'avons pas réalisé de ponction médullaire pour préciser le caractère central ou périphérique de cette thrombopénie, mais son caractère isolé laisse supposer qu'il s'agit d'une thrombopénie périphérique, et dans ce cas fort probablement immunitaire. La mesure du taux sérique d'IgG antiplaquettaires n'a pas non plus été possible faute d’équipement.

Les auteurs du case report turque décrivent une thrombopénie rebelle à la corticothérapie ainsi qu'aux perfusions d'immunoglobulines administrées [3]. Dans notre observation, la présentation clinique de la maladie était la même. Cependant, nous avons observé une excellente réponse clinique à la corticothérapie à haute dose, ainsi qu'aux transfusions de sang frais se traduisant par une majoration de la numération des plaquettes ainsi qu'une normalisation du temps de saignement après 7 jours de traitement. A ce sujet, il n'existe à ce jour aucun guideline évidence based compte tenu du faible nombre de cas observés dans la population pédiatrique. Néanmoins, des groupes d'experts de the American Society of Hematology et de the British Society for Hematology ont proposé des protocoles de prise en charge. La société Britannique propose une corticothérapie en traitement de première ligne chez l'enfant [4, 5]. Les américains préfèrent les perfusions d'immunoglobulines en première ligne et réservent l'usage des corticoïdes aux purpuras réfractaires compte tenu de leur toxicité lorsqu'ils sont administrés à long terme [5, 6].

Dans les pays à faible ressources comme la République Démocratique du Congo, la corticothérapie constitue une option efficiente compte tenu de son accessibilité financière par rapport aux immunoglobulines. Notons par ailleurs qu'il s'agit d'un traitement efficace, permettant de restaurer le taux de plaquette circulant en quelques jours; voire quelques heures. Seule une étude multicentrique randomisée bien menée, évaluant l'efficacité des différents protocoles disponibles tant en terme de qualité de vie que de cout et d'efficacité pourra mettre un terme a la controverse.

## Conclusion

Des cas de purpura thrombopénique immunologique associés à la tuberculose ont déjà décrits mais demeurent rares chez l'enfant. De plus, leur prise en charge est encore controversée. Dans les pays à faible ressources comme la République Démocratique du Congo, la corticothérapie semble constituer une option efficiente compte tenu de son accessibilité financière par rapport aux immunoglobulines. Chez notre patient, ce traitement a permis de restaurer le taux de plaquette circulant en quelques jours.
